# Significantly higher incidence of circulating fatty objects in portal blood samples compared to peripheral blood samples in patients with pancreatic ductal adenocarcinoma

**DOI:** 10.3389/fonc.2025.1611872

**Published:** 2025-09-02

**Authors:** Ruoxiang Wang, Kenneth Park, Srinivas Gaddam, Quin Liu, Rabindra Watson, Adrian Lim, Yan Ou, Yi Zhang, Mouad Edderkaoui, Michael S. Lewis, Simon K. Lo, Stephen J. Pandol

**Affiliations:** ^1^ Department of Medicine, Cedars-Sinai Medical Center, Los Angeles, CA, United States; ^2^ Department of Biomedical Sciences, Cedars-Sinai Medical Center, Los Angeles, CA, United States; ^3^ Department of Pathology, Cedars-Sinai Medical Center, Los Angeles, CA, United States; ^4^ Department of Pathology, Veterans Health Administration Greater Los Angeles Healthcare System, Los Angeles, CA, United States

**Keywords:** circulating fatty object, portal vein blood, peripheral blood, hemofiltration, pancreatic ductal adenocarcinoma

## Abstract

**Introduction:**

We previously reported the identification of circulating fatty objects (CFOs), abnormal entities found in peripheral blood samples from cancer patients. CFOs are large spherical objects with high contents of heavy cholesterol lipids, posing a risk of vessel embolization and circulation occlusion, common complications in clinical cancers. Initial characterization suggested that CFOs bear resemblance to bile salts, prompting further investigation into the potential link between bile and cancer-associated vascular occlusion.

**Methods:**

To explore this connection, we analyzed portal blood samples from patients diagnosed with pancreatic ductal adenocarcinoma (PDAC), as CFOs were most frequently detected in this type of cancer (20.4% incidence). Portal vein samples were collected via transhepatic endoscopic ultrasound-guided fine-needle aspiration and treated with ammonium chloride hemolysis to facilitate enumeration and characterization of CFOs in the nucleated blood cell fraction.

**Results:**

CFOs were observed in 14 out of the 16 portal samples, indicating a significantly higher incidence compared to peripheral samples. Despite this, portal CFOs exhibited similar characteristics to peripheral CFOs. In a parallel study, a portion of the portal samples was filtered through an Exthera’s Seraph 100 column before hemolysis, resulting in no detection of CFOs.

**Discussion:**

These findings suggest that CFOs originate from bile. It is probable that CFOs are insoluble bile lipids that have ebbed into the portal vein and subsequently shunted to the systemic circulation due to PDAC-induced hepatobiliary system abnormalities. It appears that hemofiltration can effectively remove CFOs from circulation.

## 1 Introduction

We have reported the discovery of a previously unrecognized entity in cancer patient peripheral blood samples (1). Without any previous reports in the literature, we have named this entity circulating fatty objects (CFOs). CFOs display specific physical properties and have the potential to act as emboli, causing vascular occlusion. After removing red blood cells (RBCs) and resuspending peripheral blood mononuclear cells (PBMCs), CFOs are visualized as fatty droplet-like spheres that do not disperse after long-term storage or incubation in aqueous solutions. Unlike common fatty droplets, CFOs in aqueous solutions exist as sediments with a density at least as heavy as the RBCs. Compared to primary lipids (*e.g.*, phospholipids, cholesterol, or triacylglycerols), the content of CFO is similar to cholesterol lipids, while our analysis has revealed that CFOs have a cholesterol-rich content (1). To investigate the organ of CFO origin, we monitored their incidence in clinical cancer patients.

Among 1,937 peripheral blood samples from patients individually diagnosed with various cancers (1), varying CFO counts were observed in 214 samples, giving an overall incidence of 11.05%. Notably, the presence of CFOs did not appear to be random. Specifically, 96 out of the 460 patients diagnosed with pancreatic malignancies were found to have these objects (20.43%), while much lower incidences were observed in malignancies of other organs such as the liver (11.17%), breast (9.22%), kidney (8.61%), and prostate (7.10%). When compared to the absence of CFOs in 80 healthy donors, these results suggest that the presence of CFOs in peripheral circulation is an event associated with cancer development, progression, or metastasis, with the likely origin of CFOs being insoluble bile acids or salts (1).

Although we suspected the gallbladder as the origin of CFOs, both the liver and the gallbladder are in close proximity to the pancreas and play roles in cholesterol lipid metabolism and storage. Relative to the portal vein circulation, the liver is downstream while the gallbladder is upstream. We hypothesized that this anatomical relationship could help identify the organ of CFO formation. If the gallbladder is indeed the origin, CFOs would first enter the portal blood before entering the systemic circulation. In this study, we examined CFO counts in portal blood samples from clinical patients diagnosed with pancreatic ductal adenocarcinoma (PDAC). CFOs were more frequently found in portal samples compared to peripheral blood, providing strong evidence in support of the gallbladder as the organ of CFO origin.

## 2 Materials and methods

### 2.1 Study subjects

Blood samples used in this study were obtained from 16 PDAC patients undergoing initial evaluation or treatment at Cedars-Sinai Medical Center between 2022 and 2023. The use of human samples for research was approved by the Institutional Review Board (IRB) with protocol numbers of Pro00041517, Pro00025217, and Pro00030418. Informed written consent was obtained for the use of blood samples in research.

Blood samples were obtained from the portal vein using endoscopic ultrasound guided fine-needle aspiration (EUS-FNA) through the transgastric approach, involving direct puncture of the portal vein with a 22-gauge needle. The feasibility and safety of this sampling protocol had been previously confirmed in farm pig models (2). Paired peripheral samples were also taken from the median cephalic/cubital veins. Each sample was collected in a 10-ml lavender-top Vacutainer tube (BD366643, Becton, Dickinson and Company, Franklin Lakes, NJ, USA), containing dipotassium ethylenediamine tetra acetic acid (K2 EDTA) as an anticoagulant. All samples were promptly transported to the research laboratory for CFO detection within 2 hours of collection.

### 2.2 Materials and agents

Chemicals used in this study were purchased from Sigma-Aldrich (St. Louis, MO, USA). These included Intralipid Emulsion, Oil Red O, the agents in the hemolysis buffer, ammonium chloride (NH_4_Cl), Tris(hydroxymethyl)aminomethane (Tris), and K2-EDTA; the detergents sodium dodecyl sulfate, Triton X-100, Tween 20, and Tween 80; the organic solvents ethanol, isopropanol, and tetrahydrofuran; and the sugars dextrose, maltose, and sucrose. Biotinylated antibodies to perilipin (NB110-40760) and the isotype IgG (NBP1-97078) were from Novus Biologicals (Centennial, CO, USA). Streptavidin conjugated Dynabeads were used from the Cellection Biotin Binder Kit (Thermo Fisher Scientific, Waltham, MA, USA).

### 2.3 Blood sample filtration

The miniaturized and heparinized Seraph 100 column (UHMWPE, ExThera Medical Corporation, Martinez, CA, USA) was used to filter whole blood samples. Pre-wetting was conducted by running 10 ml of saline through the column, which was positioned vertically at room temperature. An aliquot of the sample (between 2.5 ml and 7.5 ml) was then run through the column by gravity into a 15 ml conical collection tube for further processing.

### 2.4 Blood sample processing

RBCs were removed using ammonium chloride hemolysis with a sterile hemolysis buffer (150 mM NH_4_Cl, 15 mM Tris, pH7.4, and 0.1 mM EDTA). A blood sample was mixed with 10 volumes of the buffer and incubated at room temperature until complete hemolysis occurred. The sample was then centrifuged at 300×g for 10 minutes to recover the pellet. After being washed twice in phosphate buffered saline (PBS), the recovered pellet was resuspended in 2 ml of PBS, and the resuspension was spread onto a 10-cm Cellview cell culture dish (Greiner Bio-One, Monroe, NC, USA) to form a thin membrane.

### 2.5 CFO detection

The entire dish was visually examined under a low magnification microscope (20×) to check for the presence of CFOs. The CFOs were then counted, and the results were presented as CFO counts per 7.5 ml of whole blood. Subsequently, the CFOs were collected from the dish for further characterization.

### 2.6 Lipid staining

This study utilized the Oil Red O staining method, as described previously (3), to detect lipids in CFOs.

### 2.7 Membrane protein detection

This study utilized the protocol previously reported (1) for detecting membrane proteins. In brief, isolated CFOs were first stained with biotinylated antibodies targeting perilipin. The CFOs were then exposed to streptavidin-conjugated Dynabeads before being imaged under a phase contrast microscope.

### 2.8 Microscopic documentation

The same microscopes and photographic settings as previously reported (1) were used for imaging acquisition.

### 2.9 Statistical analysis

Fisher’s exact test was used. This test calculates the exact probability of obtaining a table as extreme or more extreme than the one observed, assuming the null hypothesis that the positive rate is the same in both groups. A probability of *p*<0.05 was deemed statistically significant.

## 3 Results

### 3.1 CFOs are present in the portal blood of the majority of PDAC patients

The portal vein drains blood from abdominal organs, including the pancreas and the gallbladder. Before being processed by the liver, portal blood may contain biomarkers that could be used for diagnosing PDAC. A thorough analysis and in-depth examination of portal blood samples may also shed light on the mechanism of PDAC progression and metastasis. This rationale prompted us to launch the PancBank project, which involves studying biomarkers for pancreatic diseases by collecting hepatobiliary/pancreatic tissue, peripheral and portal blood as well as bile samples for research in the realm of pancreatic disorders.

The Panc-Bank project facilitated a comprehensive examination of invaluable rare biofluids essential for mechanistic studies on PDAC progression and metastasis. For example, circulating tumor cells in PDAC patient portal blood have been genetically annotated (4), and microvesicles or oncosomes could be characterized (5). Importantly, both studies provided strong evidence that these PDAC-mediating factors could be removed by hemofiltration. The objective of the current study is to assess whether CFO counts from portal blood samples are significantly higher than those of the peripheral samples, and whether these abnormal entities can be eliminated by hemofiltration.

Portal blood samples used in this study were collected from 16 PDAC patients within a year’s time span. The inclusion of individual portal samples in this study was primarily based on availability, as multiple studies used the samples. Paired peripheral samples were obtained nearly simultaneously from 7 of these patients as the portal blood was collected through the EUS-FNA procedure. Besides blood samples and the PDAC diagnosis, no other patient information was used for the study.

**Figure 1:**
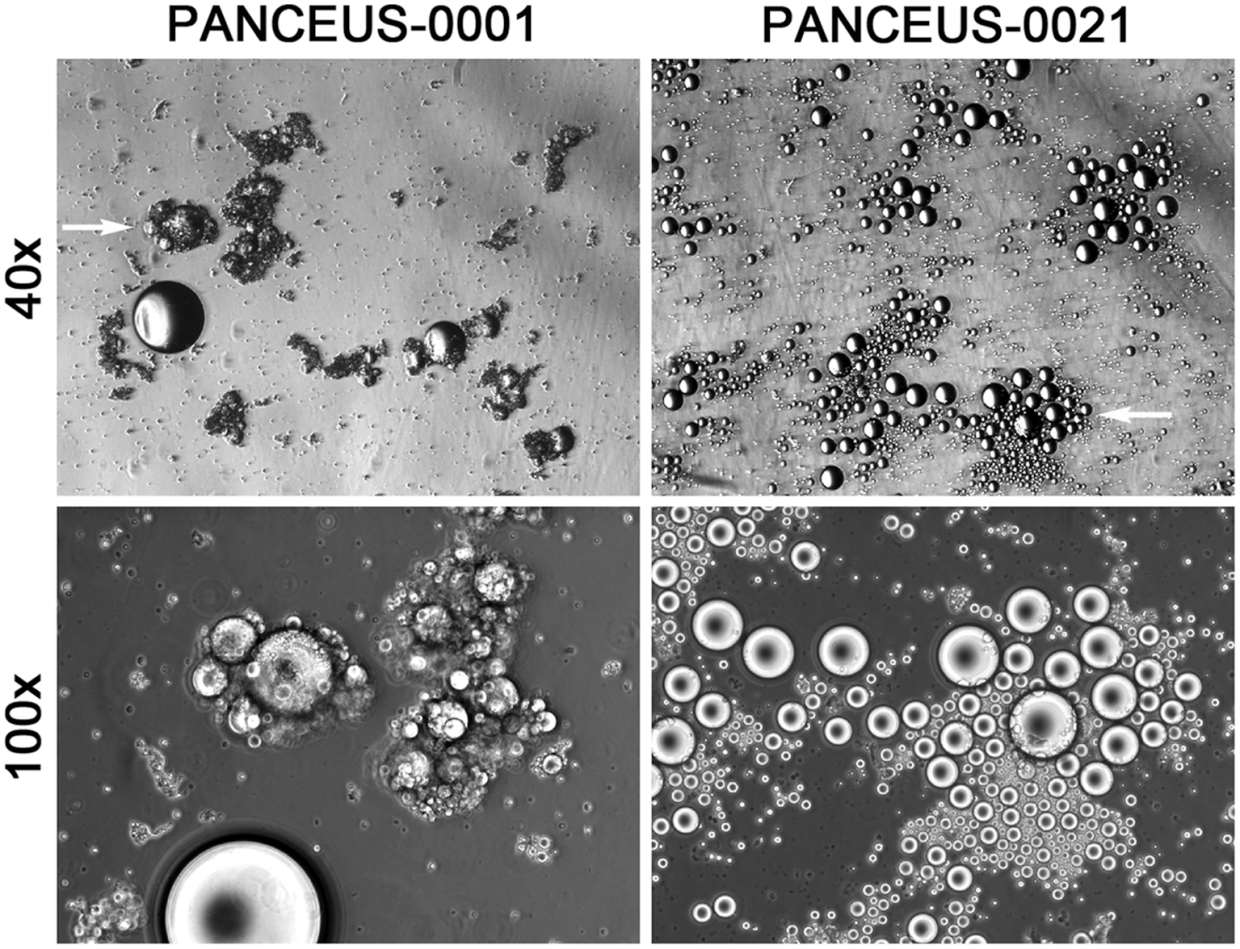
CFOs in the portal vein blood of PDAC patients. Representative results from two patients are shown. CFOs were found among PBMCs after the removal of RBCs. In the PANCEUS-0001 sample, CFOs were intertwined with fibrous materials, suggesting intravascular clotting. In the PANCEUS-0021 sample, CFOs were abundant. PBMC counts were notably low in these samples. Arrows indicate areas for higher magnification images.

**Figure 2:**
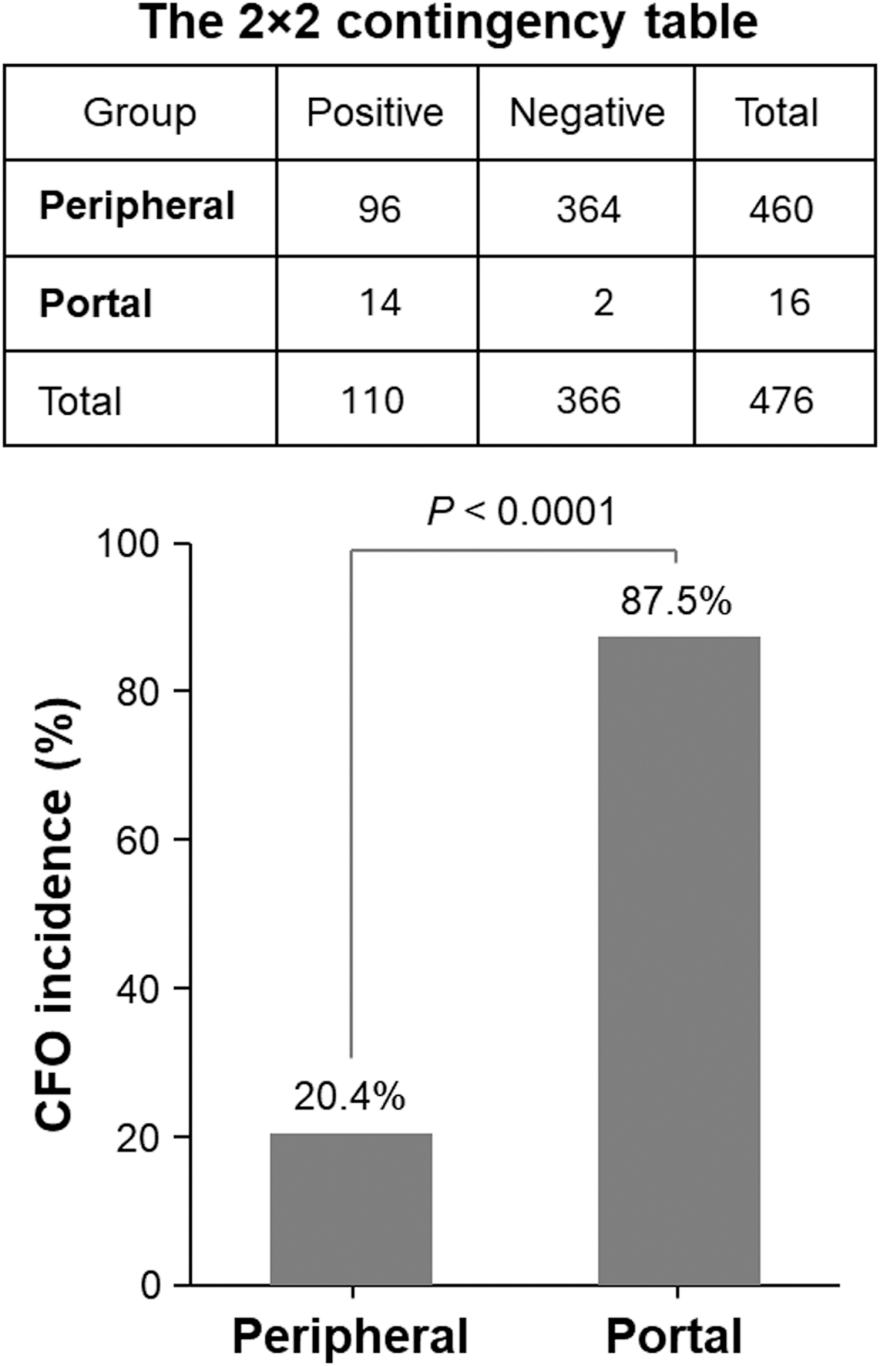
Significantly higher incidence of CFOs in portal blood samples compared to peripheral blood samples. Fisher’s exact test was used to determine the difference. The table in the top shows a summary of the CFO detection. The histogram in the bottom presents the result of statistical analysis.

This examination identified CFOs in the majority of the portal blood samples. In contrast to the scenario of CFO detection from peripheral blood, where 94 out of the 460 patients were found with CFOs (1), 14 portal blood samples out of the 16 PDAC patients were detected with CFOs (**Table 1**). After removing RBCs by ammonium chloride hemolysis, CFOs were observed in sizes ranging from 100 to over 1,000 µm in diameter in the PBMC fraction (**Figure 1**). Similar to the findings with peripheral blood samples of the PDAC patients (1), some of the CFOs in portal blood were in clusters, entangled with fiber networks, suggestive of intravascular clotting. Although CFO sizes in many portal samples were large, we considered the size to be less informative, because, during sample preparation, large CFOs might be broken into smaller ones by the force of shear or trituration. Nonetheless, this study revealed a significantly high incidence of portal CFO detection (87.5%), relative to the incidence of peripheral CFO detection (20.4%). Due to the small sample size in the portal group (n = 16), Fisher’s exact test was used in data analysis. The difference in incidence rates (20.4% vs. 87.5%) is statistically significant (*p*< 0.0001) (**Figure 2**).

**Table 1:** CFO quantification (counts/7.5 ml whole blood)^1^.

PDAC patients	Blood sample ID	Whole blood		Filtered blood^2^	
		Portal	Peripheral	Portal	Peripheral
1	PANCEUS-0001	3,600			
2	PANCEUS-0019	1,650			
3	PANCEUS-0020	3,450			
4	PANCEUS-0021	8,550			
5	PANCEUS-0022	1,350			
6	PANCEUS-0023	2,550	0		
7	PANCEUS-0024	900		0	0
8	PANCEUS-0025	0		0	0
9	PANCEUS-0026	1,350		0	0
10	PANCEUS-0027	3,750	0		
11	PANCEUS-0028	1,800	0	0	0
12	PANCEUS-0029	600	0	0	0
13	PANCEUS-0030	0	0	0	0
14	PANCEUS-0031	1,800	0		
15	PANCEUS-0032	180			
16	PANCEUS-0034	90	0		

^1.^ Blank cells indicate no samples being collected or assayed.

^2.^ Blood was filtered through Seraph 100 column and then to CFO enumeration.

### 3.2 Portal CFOs share the same characteristics as peripheral CFOs

In this PDAC cohort, 7 of the 16 PDAC patients have paired peripheral and portal vein blood collections. From these 7 patients, 6 portal vein samples had CFOs whereas none of the 7 peripheral samples contained any CFO. There is, therefore, a marked difference in CFO incidence between portal blood and peripheral blood samples.

One possible explanation for this difference is that PDAC metastasis results in the formation of CFOs, which primarily reside in the portal circulation compartment and only occasionally enter the systemic blood. Since no CFOs were found in any of the 7 peripheral blood samples, we examined the portal CFOs to confirm that they share the general characteristics as reported in our previous study. CFOs from patients 1 and 4 were used, as their portal blood samples contained sufficiently large numbers of CFOs (**Table 1**) to perform further characterization.

**Figure 3:**
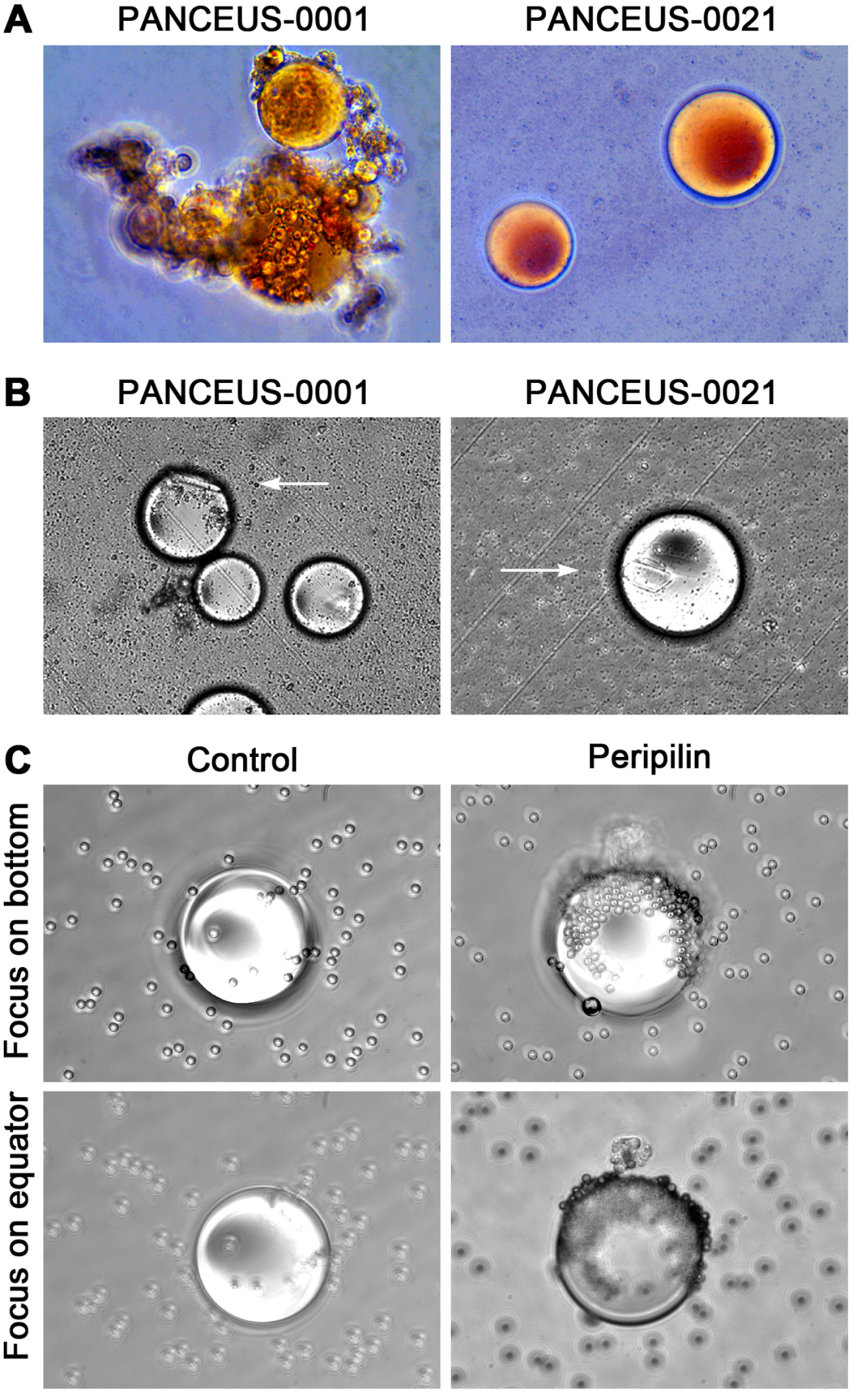
Characterization of CFOs from portal samples. Representative results from PANCEUS-0001 and PANCEUS-0021 are shown. **(A)**, Oil Red O staining indicates a lipid-rich content in the CFOs. **(B)**, cholesterol crystals (arrows) were present in many CFOs. **(C)**, in this study, a biotin-conjugated antibody was used to react with the membrane perilipin proteins on the CFOs from PANCEUS-0021. Streptavidin-conjugated Dynabeads were used to visualize the reaction. In the control group, biotin-conjugated IgG was used. To determine the binding on the surface, the CFOs were imaged by focusing on the bottom and then on the equator.

CFOs isolated from the PBMC resuspension using wide-pore pipette tips were subjected to Oil Red O staining. Consistent with the staining of peripheral CFOs (1), portal CFOs also stained positive, indicating lipid content (**Figure 3A**). Additionally, many portal CFOs observed under a phase-contrast microscope contained plate-like monohydrate crystals (**Figure 3B**), structures reminiscent of cholesterol crystallization in gallbladder bile (6, 7).

Our previous study indicated that peripheral CFOs were enveloped in a membrane decorated with perilipin (1). Using the same indirect method, we examined whether portal CFOs were also enclosed in a membrane by visualizing surface perilipin proteins through nanoparticle labeling. Staining with an isotype control did not show any presentation, but staining with anti-perilipin antibody unveiled significant nanoparticle presentation on the surface of CFOs, displaying a staining pattern similar to that observed in peripheral CFOs (**Figure 3C**).

These results suggest that portal CFOs share general characteristics with peripheral CFOs, indicating that CFOs in portal blood and peripheral CFOs may be formed by the same mechanism and originate from the same organ.

### 3.3 CFOs are highly stable

CFOs have a high density, settling in an aqueous solution, while common lipids, either in stock or as an emulsion, would float on the surface. Previous research has shown that CFO spheres can remain intact in cell culture medium for at least 8 weeks (1). To evaluate their stability, we first tested portal CFOs and found that they were not affected by detergent treatments. Even when exposed to concentrations of up to 1% (w/v) with agitation for 24 hours, sodium dodecyl sulfate, Triton X-100, Tween 20, or Tween 80 were unable to dissolve CFOs, although they were able to dissolve the control Intralipid Emulsion (Sigma-Aldrich). Secondly, CFOs did not shrink when exposed to high osmotic concentrations (50%, w/v) of dextrose, maltose, or sucrose, indicating a lack of aqueous components. Thirdly, CFOs showed resistance to solvents such as ethanol (ranging from 10% to 50%), isopropanol, and tetrahydrofuran. These results strongly suggest that CFOs are not composed of common lipids but rather specific cholesterol metabolites, most likely bile acids or bile salts.

### 3.4 Removal of CFOs by hemofiltration

We have previously reported that circulating tumor cells (4), and microvesicles or oncosomes (5) can be effectively depleted by hemofiltration through the Seraph 100 column. We wanted to test whether CFOs in the portal blood could also be removed using this technology.

To do so, we took 3.75 ml aliquots of blood samples from 6 out of the 16 PDAC patients and subjected them to hemofiltration before ammonium chloride hemolysis. In contrast to the presence of CFOs in 4 out of the 6 samples (samples 7, 9, 11, and 12, **Table 1**), no CFO was detected in any of the filtered samples. This demonstrates that CFOs can be effectively removed by hemofiltration.

## 4 Discussions

The results of our previous study have identified CFOs as an abnormal entity in the peripheral blood of many clinical cancer patients, containing heavy cholesterol lipids, probably insoluble bile acids or bile salts from the gallbladder. In the current study, we examined portal vein blood samples of PDAC patients to investigate evidence supporting the origin of CFOs from the gallbladder.

Compared to the sporadic appearance in peripheral blood samples, CFOs were present in the majority of PDAC patient portal blood samples (**Figure 1** and **Table 1**). Due to a limited sample number, no CFOs were found in the 7 paired peripheral blood samples. However, we concluded that portal CFOs and peripheral CFOs, which were characterized in the previous study, have the same organ origin. In addition to shared morphology and behavior, portal CFOs were found to have a lipid-rich content (**Figure 3A**), with cholesterol-like crystals (**Figure 3B**) and a perilipin-decorated membrane (**Figure 3C**), the same characteristics as the peripheral CFOs.

### 4.1 The difference in the incidence of CFOs between peripheral and portal blood samples

Compared to peripheral blood sampling, obtaining a portal vein sample is more invasive. In contrast to the previous study that obtained peripheral blood samples from 460 PDAC patients, this study examined portal blood samples from 16 patients. The majority (87.5%) of these samples were found to contain CFOs, and the high statistical significance (**Figure 2**) suggests that the observed difference in CFO incidence is unlikely due to random chance. This study shows that CFOs are primarily compartmentalized in portal blood and only occasionally enter systemic circulation, supporting our hypothesis that CFOs are derived from gallbladder bile, following gallbladder leakage caused by PDAC invasion and metastasis.

**Figure 4:**
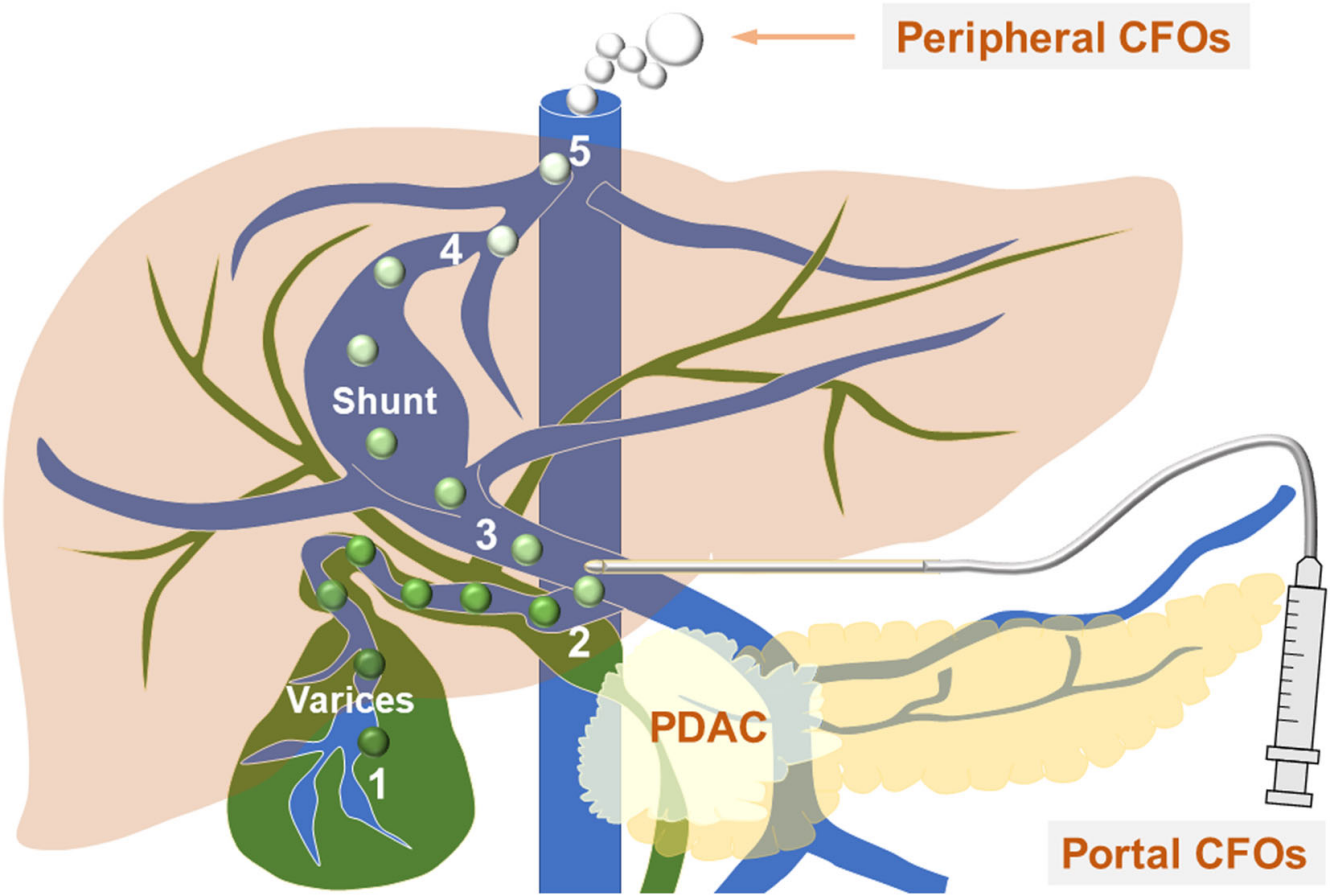
CFOs evade hepatic filtration to enter systemic circulation - the working model. The diagram shows venous vessels (blue) from both the systemic and portal systems, as well as biliary ducts (green). The process of bile entering the systemic circulation is outlined as follows: it first enters through a ruptured gallbladder wall into the variceal cystic vein blood (1), where the soluble components of the bile are diluted, and insoluble bile acids and salts condense to form CFOs. The bile then progresses to the portal vein (2), travels to the portosystemic shunt between (3) and (4), and ultimately reaches the inferior vena cava (5). Along this path, bilirubin in the bile becomes diluted into the blood as the CFOs lose their color. Within the systemic circulation, these spheres may fuse to create larger CFOs. A needle is positioned at the site of EUS-FNA.

How can CFOs evade hepatic filtration to enter systemic circulation? PDAC progression and metastasis can cause abnormalities in both the portal system and the biliary system. Invasive tumor growth results in portal hypertension and the development of various arterial-venous and portal-systemic shunts (8, 9), while hypertension may induce gallbladder cystic vein varices (10–12). In addition to damaging the blood-bile barrier, structural damage to the gallbladder wall and bile tract is common in PDAC progression and metastasis, observed in 92% of cases (13). PDAC can harm the biliary system in several ways (14–18). The tumor may constrict the biliary duct, causing bile buildup and biliary tract dilation (19–21), potentially leading to bile leakage from the gallbladder first to the cystic vein and then to the portal blood. Together, the damages in the portal system and biliary ducts create a pathway for gallbladder bile to enter the blood stream, first through the cystic veins to the portal system, and then through the shunts to the systemic circulation (**Figure 4**).

### 4.2 The potential nature of CFOs

Although CFOs have been identified as a previously unrecognized entity, bile reflux is a well-known phenomenon. Bile acids and bile salts, end products of cholesterol metabolism by the liver, are concentrated and stored in the gallbladder (22, 23). Unlike the damage of the blood-bile barrier, PDAC-induced damages in both the portal system and the biliary system may create additional pathways for bile reflux. During this process, soluble bile lipids and bilirubin disperse, while insoluble bile components condense to form CFOs. This theory is supported by the presence of cholesterol-like crystals inside the CFOs (**Figure 3B**). Interestingly, CFO-like droplets have been observed experimentally by epithelial cells of the gallbladder lumen (24, 25).

### 4.3 The potential clinical relevance of CFOs

With their chemical nature yet to be fully investigated, CFOs may have clinical relevance. The actual size of CFOs in their natural state is unknown, but isolated spheres obtained through physical processes typically range from 100 nm to 1,000 nm or even larger in diameter. CFOs have the ability to fuse with each other or with PBMCs, increasing in size (1). Due to their large size and spherical stability, CFOs can act as emboli, leading to vascular occlusion, a severe complication in the clinical progression and metastasis of PDAC. Around 94% of clinical cancer patients experience complications from vascular occlusion (26–28), which can result in debilitating and fatal ischemic conditions. Vascular occlusion is the second leading cause of cancer mortality (29–32). The exact cause of this complication is still debated. While it has been extensively researched as cancer-associated thrombosis, it remains unclear whether thrombosis or embolism is the primary factor. This is a crucial issue as the answer will determine evidence-based treatment.

Although cancer patients are believed to have hypercoagulative blood, managing cancer-associated vascular occlusion with anticoagulation therapy is challenging (33). All anticoagulation strategies (factor Xa inhibition, vitamin K antagonist, and antithrombin activation) have limited efficacy and cannot prevent the occurrence of cancer-associated vascular occlusion (33–36), which often recurs despite treatment (37–41). Additionally, the risk of occlusion is not reduced in cases of thrombocytopenia (42). If CFOs act as emboli, blood clotting would be a secondary effect, occurring only after blood flow is occluded by the CFOs.

By using the Seraph 100 column to filter 6 out of the 16 portal vein samples, we demonstrated that a single run through the column could effectively eliminate CFOs (**Table 1**), while the majority of RBCs and PBMCs were not trapped. Since CFOs are much larger than human cells, size exclusion could be a mechanism for CFO removal. Further evaluation is needed to determine if this observation suggests an alternative therapeutic strategy for cancer-associated vascular occlusion.

### 4.4 Limitations of the study

This study was conducted prospectively in a double-blind test, focusing on the presence of CFOs in PDAC patient portal blood. To identify the gallbladder as the source of CFOs, it is necessary to assess damage and leakage of the gallbladder wall, along with the pathological detection of cystic varices and arterial-venous or portal-systemic shunts.

The conclusions drawn from this study should be interpreted with caution, as the sample size of 16 PDAC patients is small. Further investigation is needed to validate the conclusion, despite the finding being determined with sufficient statistical power.

The high incidence of CFOs in PDAC patient portal blood samples supports the gallbladder origin of CFOs. However, it remains unclear whether CFOs detected in other types of cancers (1) share the same origin. It is crucial to determine if CFOs are formed with insoluble bile acids or bile salts. Both bile acids and bile salts can become extremely insoluble depending on their conjugation status, pH level, Ca^++^ concentration, and temperature (43–46). Currently, individual molecules in bile can be identified using ultrahigh performance liquid chromatography with tandem mass spectrometry (47–49). Confirming the molecular similarity between CFO and bile will establish the gallbladder as the organ responsible for CFO formation.

## Data Availability

The original contributions presented in the study are included in the article material. Further inquiries can be directed to the corresponding author.
